# A Case of Extranodal NK/T-cell Lymphoma Infiltrating Kidneys, Presenting With Hematuria and Proteinuria With False-Positive Serum Anti-proteinase 3, and Mimicking Granulomatosis With Polyangiitis

**DOI:** 10.7759/cureus.49794

**Published:** 2023-12-01

**Authors:** Pongpratch Puapatanakul, Athiphat Banjongjit, Nattachai Srisawat, Natavudh Townamchai, Talerngsak Kanjanabuch

**Affiliations:** 1 Division of Nephrology, Department of Medicine, Faculty of Medicine, Chulalongkorn University, Bangkok, THA; 2 Nephrology Unit, Department of Medicine, Vichaiyut Hospital, Bangkok, THA; 3 Renal Immunology and Renal Transplant Research Unit, Department of Medicine, Faculty of Medicine, Chulalongkorn University, Bangkok, THA; 4 Center of Excellence in Kidney Metabolic Disorders, Faculty of Medicine, Chulalongkorn University, Bangkok, THA

**Keywords:** case report, glomerulonephritis, anca, granulomatosis with polyangiitis (gpa), nk/t-cell lymphoma

## Abstract

We report a case of misdiagnosed extranodal NK/T-cell lymphoma, nasal type, mimicking granulomatosis with polyangiitis (GPA). A 30-year-old male presented with chronic non-resolving right paranasal sinusitis for two years accompanied by multiple generalized cutaneous nodules, and subnephrotic-range proteinuria. Biopsies from skin lesions and paranasal sinuses demonstrated leukocytoclastic vasculitis and necrotizing granulomatous inflammation, respectively. Serum proteinase 3 (PR3)-antineutrophilic cytoplasmic antibody (ANCA) was positive, suggesting the diagnosis of GPA based on the 2022 American College of Rheumatology/European Alliance of Associations for Rheumatology Classification Criteria for GPA. A kidney biopsy was not pursued due to the cause of glomerulonephritis (GN) being clinically evident, per the KDIGO 2021 GN Clinical Practice Guidelines. Immunosuppression was administered, which led to a transient improvement in clinical symptoms. However, subsequent kidney biopsy and other organ biopsies with cytogenic and molecular tests eventually confirmed extranodal NK/T-cell lymphoma infiltrating kidneys, skin, and paranasal sinuses. Physicians should always consider the possibility of hematologic malignancy in young adults presenting with multiple-organ involvement with vasculitic lesions or pauci-immune crescentic GN, albeit positive ANCA serologies. Kidney biopsy with cytogenic support should be performed to exclude threatening diseases, especially in atypical cases such as in young patients despite a context of compatible manifestations with other syndromes.

## Introduction

Glomerulonephritis (GN) is a syndrome of proteinuria and glomerular hematuria, resulting from glomerular inflammation. It typically arises from various etiologies and can be classified into primary and secondary GN, each further divided into three subgroups based on immunopathologic features: (1) immune complex-mediated GN, (2) anti-glomerular basement membrane (anti-GBM) disease, and (3) anti-neutrophil cytoplasmic antibody (ANCA)-associated GN, characterized by ANCA-associated vasculitis (AAV) affecting the glomeruli, often displaying a pauci-immune necrotizing crescentic GN. AAV comprises a group of diseases characterized by systemic vasculitis affecting small- and medium-sized blood vessels and the presence of circulating ANCA, which includes granulomatosis with polyangiitis (GPA), microscopic polyangiitis, and eosinophilic granulomatosis with polyangiitis (EGPA) [1].

Although the gold standard for establishing ANCA-associated GN is a kidney biopsy, it could be overlooked in typical cases involving patients older than 50 years presenting with rapidly progressive GN or systemic vasculitis and having a positive serum ANCA [either against myeloperoxidase (MPO) or proteinase 3 (PR3)] [1]. However, non-vasculitis conditions (e.g., systemic lupus erythematosus, endocarditis, and hepatitis infections) can lead to a false-positive ANCA blood test [2,3]. Lymphoma, a hematologic malignancy typically originating from the lymphatic system, can sometimes initially present outside the lymph nodes, a condition known as primary extranodal lymphoma [4]. A variant of this condition, extranodal NK/T-cell lymphoma, shares some specific features with non-severe AAVs (limited stage), including upper respiratory tract (URT) involvement, presence of constitutional symptoms, and responsiveness to steroid and cyclophosphamide [5].

Two cases of NK/T-cell lymphoma have been reported to be misdiagnosed as AAV, limited stage. However, both these cases had no kidney involvement and negative ANCA tests [5,6]. The need to distinguish true pauci-immune necrotizing crescentic GN from its potential mimickers is one of the most challenging diagnostic conundrums in clinical medicine. We report a case of extranodal NK/T cell lymphoma presenting with GN, with URT as well as skin involvement, constitutional symptoms, and false-positive PR3-ANCA, mimicking AAVs and ANCA-associated GN, which caused a delay in the diagnosis of the lymphoma. The patient succumbed to the condition rapidly before chemotherapy could be adequately administered.

This article was previously presented as a meeting abstract at the 2023 World Congress of Nephrology (WCN) on August 4-6, 2023.

## Case presentation

A 30-year-old, previously healthy male presented to the hospital with chronic non-resolving right cheek swelling for over two years. Two years prior, he had developed fullness in both ears and progressive swelling on the right cheek. A sinoscopic biopsy of his maxilla had demonstrated chronic inflammation, and chronic maxillary sinusitis had been diagnosed. A year before the current visit, he had observed acne-like painless eruptions spreading over his face, arms, and trunk, along with a non-resolving swelling on his right cheek. He also had a low-grade fever, experienced foamy urine, and had lost 15 kilograms of body weight.

**Figure 1:**
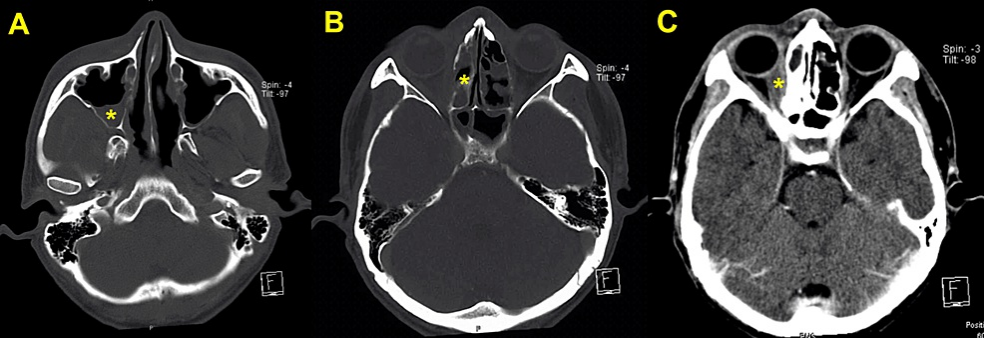
Paranasal CT scan of the patient (A) Mucoperiosteal thickening and air-fluid level in the right maxillary sinus (asterisk). (B) Mucoperiosteal thickening of right ethmoid sinuses (asterisk). (C) Diffuse haziness of extraconal and medial side of intraconal fat of right orbit (asterisk) CT: computed tomography

A paranasal CT scan revealed chronic right pansinusitis with inflammatory changes in the right orbital cavity (Figure 1). Biopsies of the skin lesions and paranasal sinuses demonstrated leukocytoclastic vasculitis (LCV) and necrotizing granulomatous inflammation, respectively. Urine analysis was remarkable for protein 2+ (normal value: negative), leukocytes of 10-20/HPF (normal value: 0-2/HPF), and erythrocytes of 50-100/HPF (normal value: 0-2/HPF). The 24-hour urine protein value was 2.3 g, and the serum creatinine value was 1.2 mg/dL. Panel serologies were performed, and only cytoplasmic (C)-ANCA and anti-proteinase 3 (PR3) were found to be positive. Normal serum complements were found. According to the 2022 American College of Rheumatology/European Alliance of Associations for Rheumatology Classification Criteria for GPA, the patient fulfilled the criteria for PR3 ANCA-associated GPA (12 points, above the cut-off 5 points) [7]. ANCA-associated GN was suspected. Treatment with oral cyclophosphamide and high-dose corticosteroids was started without diagnostic confirmation by kidney biopsy according to the KDIGO 2021 Clinical Practice Guideline for the Management of Glomerular Diseases [1].

The proteinuria and active urine sediments subsided gradually and so did the skin lesions and sinusitis. However, once the steroids and immunosuppressants were tapered off, the patient developed generalized multiple skin nodules over his arms and legs, two of which later turned into large necrotic ulcers on his left thigh and right wrist, accompanied by recurrent low-grade fever. Multiple shallow ulcers and whitish patches were observed on the posterior pharynx. Serum creatinine value bounced to 1.5 mg/dL with a high lactate dehydrogenase (LDH) of 1,513 U/L. Urine examination again revealed active sediment (leukocytes: 10-20/HPF, erythrocytes: 50-100/HPF) with dysmorphic microscopic hematuria and 1+ proteinuria (2 gm of quantitative proteinuria) (Table 1).

**Table 1:** Lab investigations during presentation with skin and pharynx ulcers Anti-PR3: anti-proteinase 3; c-ANCA: cytoplasmic antineutrophil cytoplasmic antibodies; LDH: lactic dehydrogenase

Parameters	Patient value	Reference range
Serum creatinine, mg/dL	1.5	0.50-1.00
Urinalysis dipstick		
Protein	1+	Negative
WBC, cells/HPF	10-20	0-2
RBC, cells/HPF	50-100	0-2
c-ANCA	Positive	Negative
Anti-PR3	Positive	Negative
LDH, U/L	1,513	230-460

**Figure 2:**
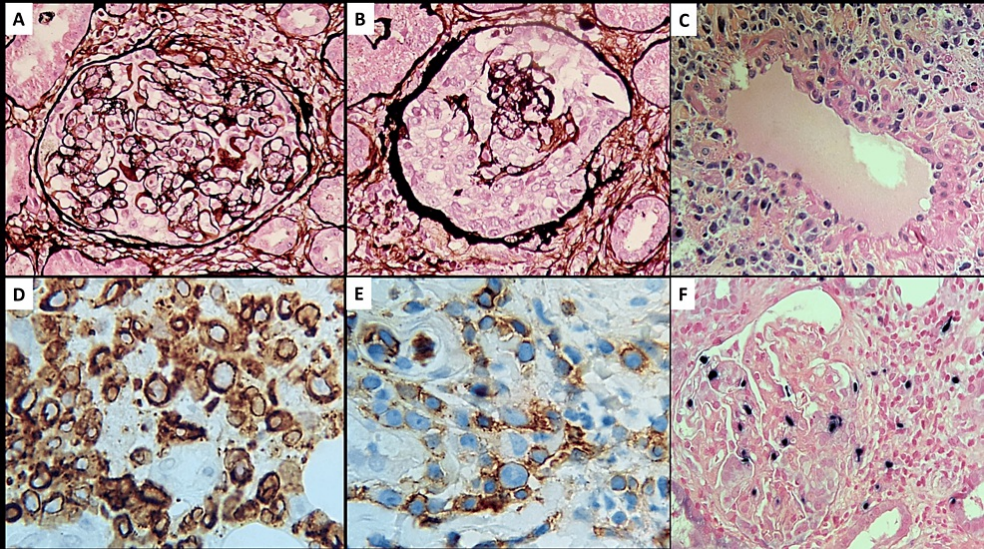
Histopathologic examination The examination showed mild mesangial proliferation with focal endocapillary mononuclear cell infiltration (A, Jones Silver stain 400x), crescent formation (B, Jones Silver stain 400x), and perivascular cell infiltration with medium to large atypical lymphocytes (irregular nuclear folding with a granular appearance) (C, Hematoxylin & Eosin stain x200). The infiltrated cells stained positive for CD3 (D, cytoplasmic pattern), CD56 (E), and EBER (F) EBER: Epstein-Barr virus-encoded small RNAs

Several intraabdominal and intrathoracic sub-centimeter lymph nodes of up to 0.9 cm in size were detected on CT imaging. A kidney biopsy revealed 14 glomeruli, including two globally sclerosed glomeruli. All of the non-sclerosed glomeruli revealed mild widening of the mesangial area with a mild increase in mesangial cellularity. No endocapillary hypercellularity was present. Two glomeruli contained crescents (Figures 2A, 2B). There was no evidence of fibrinoid necrosis or arteritis besides perivenular cell infiltration (Figure 2C). However, dense infiltration of medium-sized atypical mononuclear cells was found in the glomerular tuff and crescentic lesions (Figure 2F). Immunofluorescence exhibited granular polyclonal Ig, C3, kappa, and lambda deposits (1-2+). Immunohistochemistry revealed CD3+ (a marker of T-lymphocyte), CD56+ (a marker of NK cell), negative CD20 (a marker of B cell), and positive Epstein-Barr virus (EBV)-encoded small RNAs (EBERs) in all biopsied kidney structures, including in the crescents (Figures 2D-2F). In situ hybridization for EBERs showed positive staining on infiltrating lymphocytes for all available clinical samples, including ethmoid/maxillary sinuses, skin, and kidney specimens. The immunophenotypic histopathology of lymphocytic infiltrations and evidence of prior infection by EBV fulfilled the diagnosis criteria for extranodal Nk/T cell lymphoma. On day 49 after the onset of the treatment, during a course of systemic chemotherapy, the patient passed away from invasive pulmonary aspergillosis. The first skin and paranasal sinus biopsies that were obtained before immunosuppression were retrospectively immunohistochemistry-stained, and they revealed CD3+, CD5-, CD4-, CD8+/-, BF1-, EBER+, CD56-/+, TIA1+, granzyme B+, CD30+, ALK-, CD20-, Ki67+ (high index), monoclonal TCR-B, monoclonal TCR-G, and no monoclonal TCR-D, indicating that the patient had NK/T cell lymphoma from the beginning itself.

## Discussion

We described a misdiagnosed case of extranodal NK/T-cell lymphoma, nasal type, mimicking GPA; both diseases share several similarities in clinical manifestations, tissue pathologies, and positive ANCA serologies. The diagnosis was confirmed after cytogenic and molecular tests were applied to the tissue specimens. The presence of NK/T-cell lymphoma in the crescents suggests lymphoma infiltration as the cause.

Kidney infiltrations by B-cell lymphoma have been occasionally reported as causing proteinuria (65%), hematuria (41%), and elevated serum creatinine [8], while T-cell lymphoma infiltrating the kidneys has been rarely reported in the literature [9]. Extranodal NK/T-cell lymphoma is an aggressive non-Hodgkin lymphoma of putative NK-cell origin and is significantly associated with EBV infection [10]. Hence, the obligatory diagnostic criteria include the presence of EBER transcripts on lymphoma cells that express either CD56 or cytotoxic molecules (e.g., granzyme). While this lymphoma has been reported worldwide, it shows a predilection for Asian and Latin American populations [11]. In approximately 80% of cases, URT is the primary presentation site, and this variant is defined as the nasal subtype [11]. It is classified as a disseminated subtype with a dismal outcome if the lesion extends beyond the primary area or involves the bone marrow, as demonstrated in the presented case [11]. The five-year overall survival is 40% compared to >60% in the limited nasal stage [11]. Another, uncommon variant, defined as a non-nasal subtype, primarily involves organs other than the URT, including skin, and the gastrointestinal tract [11].

In our patient, the golden periods for chemotherapy were missed twice due to standard tissue pathology without cytogenic and molecular supports, while the disease had been in the limited (two years PTA) and early disseminated stages (one year PTA), resulting in delayed treatment. The lymphoma sometimes demonstrates angiocentric features [12,13], as observed in the skin biopsy (LCV) and the second sinus biopsy (necrotizing granulomatous inflammation). The disease was complicated by false-positive results of ANCA serologies, with both screening (indirect immunofluorescence technique) and confirmative (ELISA) methods [3]. False-positive p-ANCA and c-ANCA serologies have been reported in lymphoma in 3% and 1% of cases, respectively, albeit none of these were confirmed by ELISA tests [14].

Although our patient had clinical manifestations, the laboratory results and tissue pathologies fulfilled the criteria for the diagnosis of GPA and he had a favorable response to therapy initially. There are several clues that help guide the diagnosis. Firstly, the age of the patient at the time of onset was not compatible with ANCA-associated GN since AAVs commonly involve people in late middle age (50s and 60s). Secondly, several Ig and C3 deposits were detected in the glomeruli on the immunofluorescence study, which are usually trace or negative deposits in the ANCA-associated GN [15]. However, the coexistence of NK/T-cell lymphoma and GPA, or the possibility that NK/T cell lymphoma triggered GPA, cannot be fully excluded.

In our patient, the diagnosis of extranodal NK/T-cell lymphoma was established by positive CD3+ CD56+, CD20-, and EBER+ on infiltrating lymphocytes in all tissue specimens collected during the first and second presentations. Physicians should always consider the possibility of extranodal NK/T-cell lymphoma in young adults presenting with multiorgan involvement with vasculitic lesions, albeit positive ANCA serologies. The diagnosis of NK/T-cell lymphoma can often be missed on conventional pathologic assessment, especially when the sampled tissue has a paucity of cells due to their containing voluminous necrosis or a prominent granuloma. Cytogenic tests and EBER are helpful in the diagnosis of NK/T-cell lymphoma.

## Conclusions

Although the clinical manifestations may fulfill the criteria for ANCA-associated GN in patients presenting with glomerular hematuria and proteinuria, physicians should be mindful of other mimicking diseases such as lymphoma, particularly in young patients. This case report also highlights the important role of kidney biopsy for a proper diagnosis, especially in the context of manifestations compatible with other syndromes.

## References

[1] (2021). KDIGO 2021 clinical practice guideline for the management of glomerular diseases. Kidney Int.

[2] Geetha D, Jefferson JA (2020). ANCA-associated vasculitis: Core Curriculum 2020. Am J Kidney Dis.

[3] Shirai T, Takahashi R, Tajima Y (2009). Peripheral T cell lymphoma with a high titer of proteinase-3-antineutrophil cytoplasmic antibodies that resembled Wegener's granulomatosis. Intern Med.

[4] Thomas AG, Vaidhyanath R, Kirke R, Rajesh A (2011). Extranodal lymphoma from head to toe: part 1, the head and spine. AJR Am J Roentgenol.

[5] Lee YT, Chang YS, Lai CC, Chen WS, Yang AH, Tsai CY (2012). Natural killer (NK)/T-cell lymphoma mimicking granulomatosis with polyangiitis (Wegener's). Scand J Rheumatol.

[6] Strouse J, Rajan A, Chang HB, Dimachkie MD, Halbur C, Bettendorf B (2022). Clinical images: extranodal natural killer/T cell lymphoma as a rare mimicker of granulomatosis with polyangiitis. Arthritis Rheumatol.

[7] Robson JC, Grayson PC, Ponte C (2022). 2022 American College of Rheumatology/European Alliance of Associations for Rheumatology classification criteria for granulomatosis with polyangiitis. Ann Rheum Dis.

[8] Corlu L, Rioux-Leclercq N, Ganard M, Decaux O, Houot R, Vigneau C (2019). Renal dysfunction in patients with direct infiltration by B-cell lymphoma. Kidney Int Rep.

[9] Matsuda K, Fukami H, Saito A (2018). Rapidly progressive renal failure due to tubulointerstitial infiltration of peripheral T-cell lymphoma, not otherwise specified accompanied by uveitis: a case report. BMC Nephrol.

[10] Kwong YL (2011). The diagnosis and management of extranodal NK/T-cell lymphoma, nasal-type and aggressive NK-cell leukemia. J Clin Exp Hematop.

[11] Tse E, Zhao WL, Xiong J, Kwong YL (2022). How we treat NK/T-cell lymphomas. J Hematol Oncol.

[12] Parker NP, Pearlman AN, Conley DB, Kern RC, Chandra RK (2010). The dilemma of midline destructive lesions: a case series and diagnostic review. Am J Otolaryngol.

[13] Suzuki R (2012). NK/T-cell lymphomas: pathobiology, prognosis and treatment paradigm. Curr Oncol Rep.

[14] Cil T, Altintas A, Isikdogan A, Batun S (2009). Prevalence of antineutrophil cytoplasmic antibody positivity in patients with Hodgkin's and non-Hodgkin lymphoma: a single center experience. Int J Hematol.

[15] Fogo AB, Lusco MA, Najafian B, Alpers CE (2016). AJKD Atlas of Renal Pathology: pauci-immune necrotizing crescentic glomerulonephritis. Am J Kidney Dis.

